# Supplementary material for: Co‑cultivation of the anaerobic fungus Caecomyces churrovis with Methanobacterium bryantii enhances transcription of carbohydrate binding modules, dockerins, and pyruvate formate lyases on specific substrates
Source: Biotechnol Biofuels. 2021 Dec 10;14:234. doi: 10.1186/s13068-021-02083-w (PMC8665504; doi:10.1186/s13068-021-02083-w)
Supplement: Supplementary file 1 — Additional file 1. Additional tables and figures. [file 13068_2021_2083_MOESM1_ESM.docx]

**Supplemental Information**

**Supplementary Table 1.** The number of proteins identified as cellulases, hemicellulases, and other accessory enzymes for six sequenced anaerobic fungi annotated from genome sequencing (see methods).

|  | Number of protein Ids | | | | | |
| --- | --- | --- | --- | --- | --- | --- |
| **Hemicellulases** | *C. churrovis* | *N. californiae* | *A. robustus* | *P. finnis* | *N. lanati* | *O.* sp*. C1A* |
| GH11 | 33 | 24 | 29 | 36 | 90 | 45 |
| GH43 | 25 | 43 | 18 | 14 | 48 | 31 |
| GH10 | 12 | 58 | 15 | 21 | 59 | 32 |
| GH39 | 7 | 9 | 5 | 2 | 8 | 3 |
| GH30 | 4 | 4 | 2 | 1 | 4 | 3 |
| **Accessory enzymes** |  |  |  |  |  |  |
| Carbohydrate esterase (SGNH hydrolase domains) | 51 | 68 | 39 | 23 | 79 | 39 |
| Pectin Lyase | 53 | 127 | 44 | 49 | 142 | 72 |
| Polysaccharide deacetylase | 43 | 93 | 49 | 44 | 96 | 48 |
| Rhamnogalacturonate lyase | 2 | 9 | 3 | 2 | 12 | 1 |
| Pectinesterase | 1 | 15 | 5 | 5 | 16 | 8 |
| Glycosyl Hydrolase 88 | 0 | 2 | 0 | 1 | 2 | 0 |
| **Cellulases** |  |  |  |  |  |  |
| GH9 | 10 | 14 | 9 | 12 | 14 | 13 |
| GH6 | 18 | 27 | 12 | 21 | 89 | 49 |
| GH45 | 20 | 28 | 14 | 15 | 28 | 16 |
| GH48 | 7 | 21 | 7 | 13 | 23 | 14 |
| GH1 | 7 | 16 | 7 | 10 | 19 | 10 |
| GH5 | 23 | 65 | 26 | 26 | 70 | 47 |
| GH3 | 10 | 53 | 15 | 15 | 58 | 18 |
| GH16 | 8 | 19 | 11 | 6 | 19 | 6 |
| GH8 | 1 | 2 | 2 | 1 | 2 | 1 |
| GH31 | 3 | 10 | 7 | 2 | 11 | 19 |
| **Total** | 338 | 706 | 319 | 319 | 889 | 475 |

**Supplementary Table 2**. Co-cultivation of *C. churrovis* with *M. bryantii* induces transcriptional upregulation of genes that appear to encode proteins homologous to prokaryotic substrate binding proteins (SBPs), as well as class C G-protein coupled receptors (GPCRs). Among regulated transcripts are sequences encoding G-protein coupled receptors (7tm_3, PF00003) and sequences encoding putative substrate binding proteins (SBP_Bac_1, PF01547; SBP_Bac_3, PF00497; and SBP_Bac_8, PF13416). The table lists transcriptional regulation of genes encoding proteins that have at least one predicted transmembrane segment. Approximately half of all sequences contain at least one Pfam hit. Conditions indicate the substrate on which cultures were grown, followed by whether *C. churrovis* transcripts were upregulated or downregulated in the co-culture condition relative to fungal monocultures.

| **Condition** | **Number of transcripts affected** | **Transcripts with at least 1 Pfam hit** | **GPCR** | **SBP** |
| --- | --- | --- | --- | --- |
| Glucose Upregulated | 149 | 77 | 1 | 0 |
| Glucose Downregulated | 203 | 123 | 5 | 21 |
| Fructose Upregulated | 87 | 48 | 1 | 0 |
| Fructose Downregulated | 66 | 33 | 1 | 5 |
| Avicel Upregulated | 321 | 183 | 21 | 41 |
| Avicel Downregulated | 22 | 15 | 0 | 1 |
| Xylan Upregulated | 175 | 101 | 15 | 8 |
| Xylan Downregulated | 278 | 164 | 1 | 2 |
| Reed Canary Grass Upregulated | 44 | 17 | 0 | 0 |
| Reed Canary Grass Downregulated | 18 | 13 | 0 | 0 |

**Supplementary Table 3.** One or more genes within the *C. churrovis* genome aligned to all listed hydrogenosomal enzymes for *N. lanati*, with a %identity cutoff of 70.0 and a %subject coverage cutoff of 80.0, with the exception of the complex 2 subunit D, which only had a %subject coverage of 59.0 (%identity was 79.6). A=Avicel^®^, R=reed canary grass, G=glucose, F=fructose, X=xylan

| *C. churrovis* Protein Id | Enzyme | Upregulated or Downregulated (Substrate) | | | |
| --- | --- | --- | --- | --- | --- |
| 428490 | PFL1/PFL2 | | Upregulated (X, A, G, F) | |  |
| 11340 | PFL1/PFL2 | | Upregulated (X) Downregulated (A) | |  |
| 193710 | PFL1/PFL2 | | Upregulated (X) Downregulated (R) | |  |
| 193705 | PFL1/PFL2 | | Upregulated (X) Downregulated (R) | |  |
| 420504 | PFL1/PFL2 | |  | |  |
| 107174 | PFL1/PFL2 | | Upregulated (X, G) | |  |
| 417119 | PFL1/PFL2 | |  | |  |
| 621094 | PFL1/PFL2 | | Upregulated (X) | |  |
| 431187 | PFL1/PFL2 | | Upregulated (X) | |  |
| 214975 | PFL1/PFL2 | | Upregulated (X, F) | |  |
| 621093 | PFL1/PFL2 | | Upregulated (X) | |  |
| 277171 | PFL1/PFL2 | | Upregulated (X) | |  |
| 622192 | PFL1/PFL2 | | Upregulated (X) Downregulated (G) | |  |
| 277169 | PFL1/PFL2 | | Upregulated (X) | |  |
| 418039 | PFL1/PFL2 | | Upregulated (X) | |  |
| 416923 | PFL1/PFL2 | | Upregulated (X) | |  |
| 537129 | PFL1/PFL2 | | Upregulated (X) | |  |
| 635526 | PFL1/PFL2 | |  | |  |
| 413357 | PFL1/PFL2 | |  | |  |
| 572185 | PFL1/PFL2 | |  | |  |
| 462330 | PFL1/PFL2 | |  | |  |
| 454624 | Ac:SucCoA trans. | |  | |  |
| 454624 | Ac:SucCoA trans. | |  | |  |
| 453440 | SucCoA syn. Sub. A | |  |  |  |
| 519176 | SucCoA syn. Sub. B | |  |  |  |
| 526974 | SucCoA syn. Sub. B | |  |  |  |
| 243125 | Hydrogenase 1 | | Downregulated (R, G) |  |  |
| 456067 | Hydrogenase 2 | |  |  |  |
| 557447 | Complex 1: nuoF | | Downregulated (G) |  |  |
| 454874 | Complex1: nuoE | | Downregulated (X, G) |  |  |
| 452336 | Complex 2: sub. A | |  |  |  |
| 416671 | Complex 2: sub. B | |  |  |  |
| 544208 | Complex 2: sub. C | |  |  |  |
| 417861 | Complex 2: sub. D | |  |  |  |
| 549900 | Fumarase | |  |  |  |
| 487810 | ATP syn.: sub. Alpha | |  |  |  |
| 443140 | ATP syn: sub. Beta | |  |  |  |
| 459763 | ATP syn: sub. Delta | |  |  |  |
| 523564 | ATP syn.: sub. Gamma | | Upregulated (G) |  |  |

**
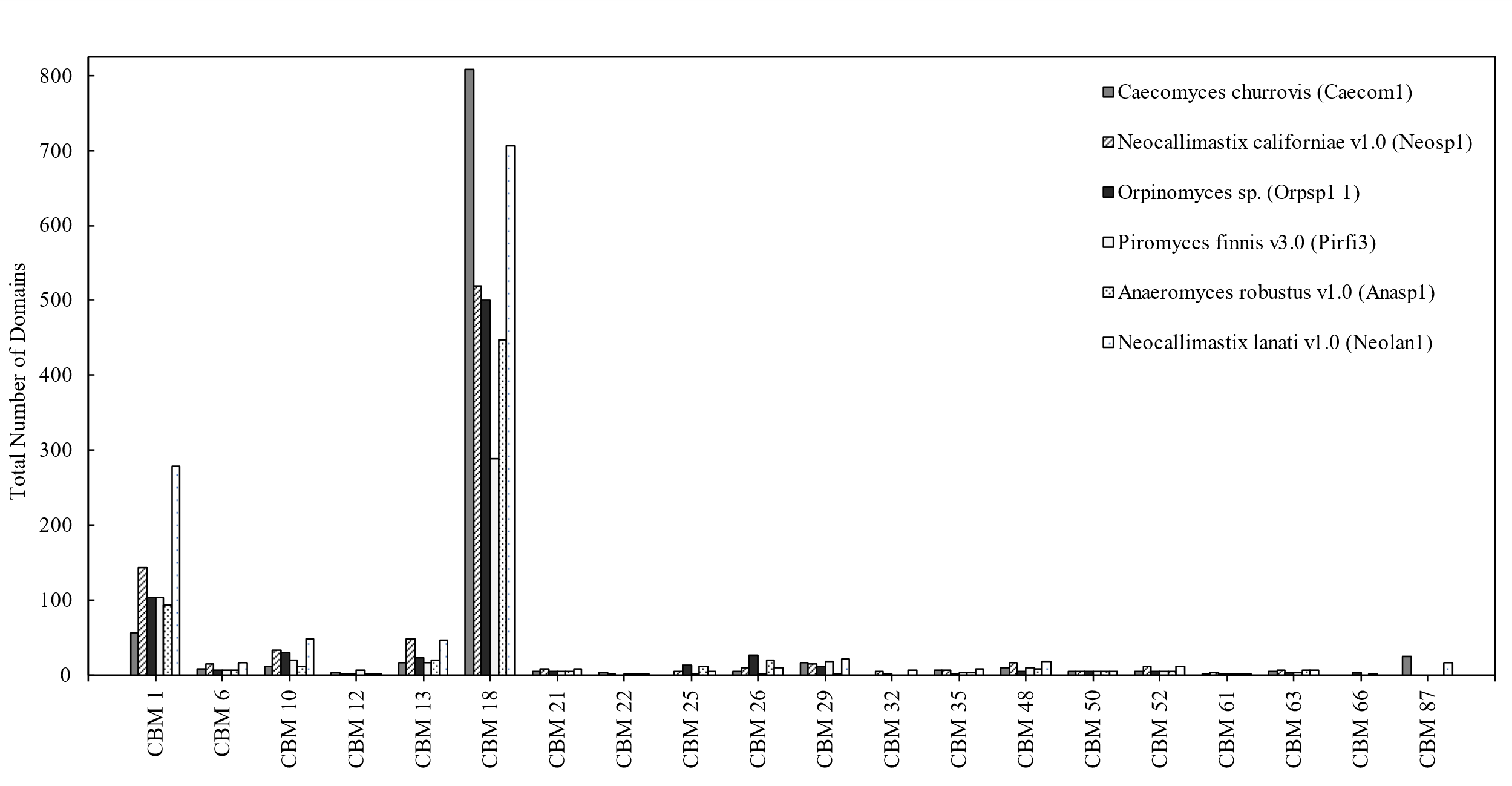
**

**Supplementary Figure 1.** *C.* *churrovis* has the highest number of CBM family 18 domains among sequenced anaerobic fungi to date.

|  |  |  |  |
| --- | --- | --- | --- |
|  |  |  |  |


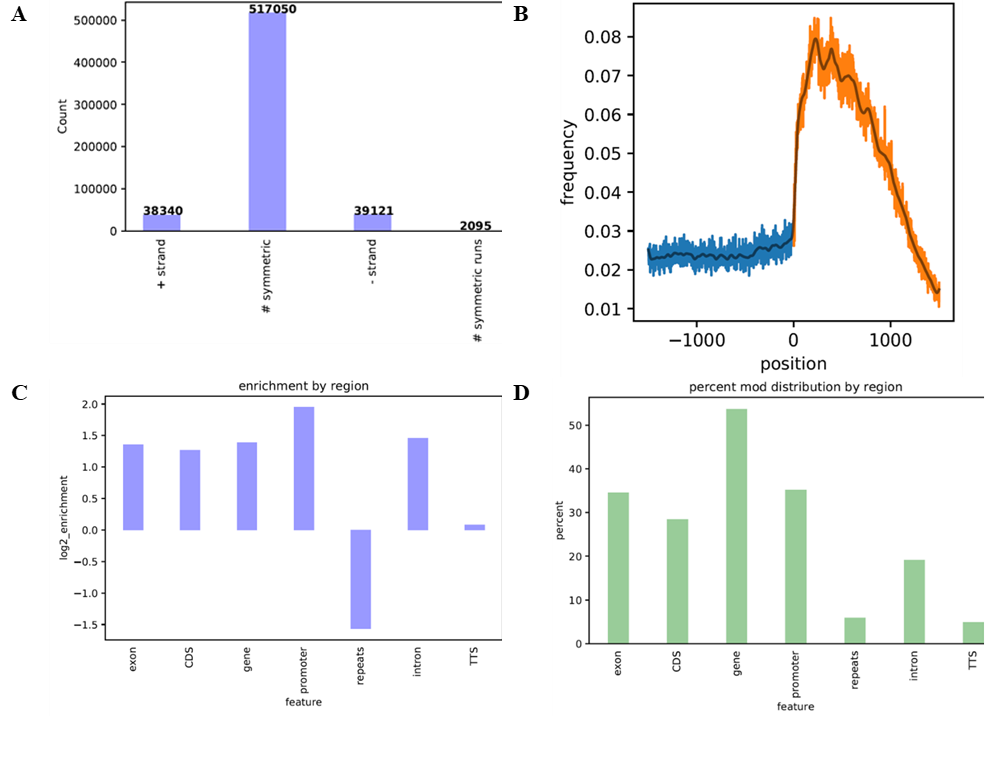


**Supplementary Figure 2. 6mA Modifications occur symmetrically at ApT dinucleotides and are concentrated in methylated adenine clusters (MACs) surrounding the transcriptional start sites of expressed genes.** This finding agrees with previous work examining 6mA modification in 16 other fungal genomes, including other early-diverging fungi. As shown in Panel A, 92.2% of modifications were symmetric within AT context and 83.32% of all modifications were symmetric. In addition, 89.67% of modifications were in AT context. Panel B shows the frequency (# 6mA observed / # available sites) per position ±1500 bp surrounding transcriptional start sites in *C. churrovis*. A slight wave in modification frequency is observed following the start of the 5' utr, not seen before in other early-diverging fungi, but the presence of modifications at the start of genes is in agreement with previous studies of fungal 6mA modifications. Panel C shows the log2fold enrichment of 6mA modifications by region. Log2_enrichment refers to the enrichment of 6mA at a given feature relative to the expected abundance of 6mA genome-wide, normalized by GC content. Panel D shows the percent of total 6mA modifications that are found within each region. Note that many of these regions overlap, such as introns and genes. Regions are defined as follows: exons – non-intron genic space, CDS – coding sequence only, gene – entirety of genic space, promoter – ±500bp surrounding transcriptional start sites, repeats – repetitive sequences identified using RepeatScout and RepeatMasker, intron – introns within genic regions, TTS – ±250bp surrounding transcription termination sites.

**
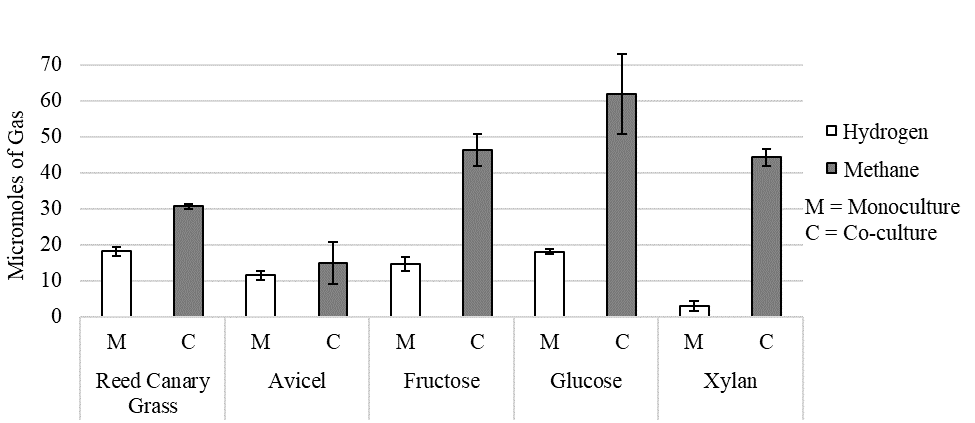
**

**Supplementary Figure 3.** End-point methane and hydrogen measurements for monocultures of *C. churrovis* and co-cultures of *C. churrovis* and *M. bryantii*. Gas chromatography was used to determine the concentration of methane and hydrogen in the headspace gas of co-cultures and monocultures on each substrate upon harvest for RNA extraction. No significant amount of hydrogen was detected in the co-cultures, and no methane was detected in the monocultures. Significantly higher amounts of methane were produced on soluble substrates.

**
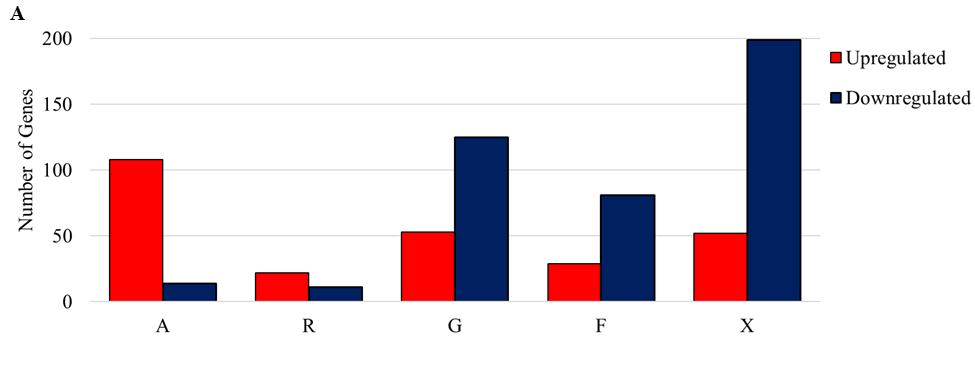
**

**Supplementary Figure 4.** The total number of genes annotated as CAZymes upregulated or downregulated overall (A) and differentially regulated genes (B-D) annotated as carbohydrate binding module (CBM), glycoside hydrolase (GH), carbohydrate esterase (CE), polysaccharide lyase (PL), and glycosyltransferase (GT) families for fungal-methanogen co-cultures of *C. churrovis* paired with *M. bryantii* relative to fungal monocultures of *C. churrovis* on a range of substrates. A=Avicel^®^, R or RCG=reed canary grass, G=glucose, F=fructose, X=xylan, Y=yes, N=no.

|  |  |
| --- | --- |
|  |  |
|  | |
|  | |
|  | |

**Supplementary** **Figure 5. Differentially regulated genes annotated as CBM, GT, PL, CE, and GH families or containing dockerin domains in three fungal strains in fungal-methanogen co-culture versus fungal monoculture on a reed canary grass substrate.** CBM 1 and CBM 18 families were significantly regulated (number of genes regulated > or =5). The CBM 1 family was highly upregulated in the *N. californiae* strain. GH 3 and GH 5 were significantly downregulated in the *N. californiae* strain (number of genes > or =5). GH=glycoside hydrolase, GT=glycosyltransferase, PL=polysaccharide lyase, CBM=carbohydrate binding module, CE=carbohydrate esterase, DOC=dockerin


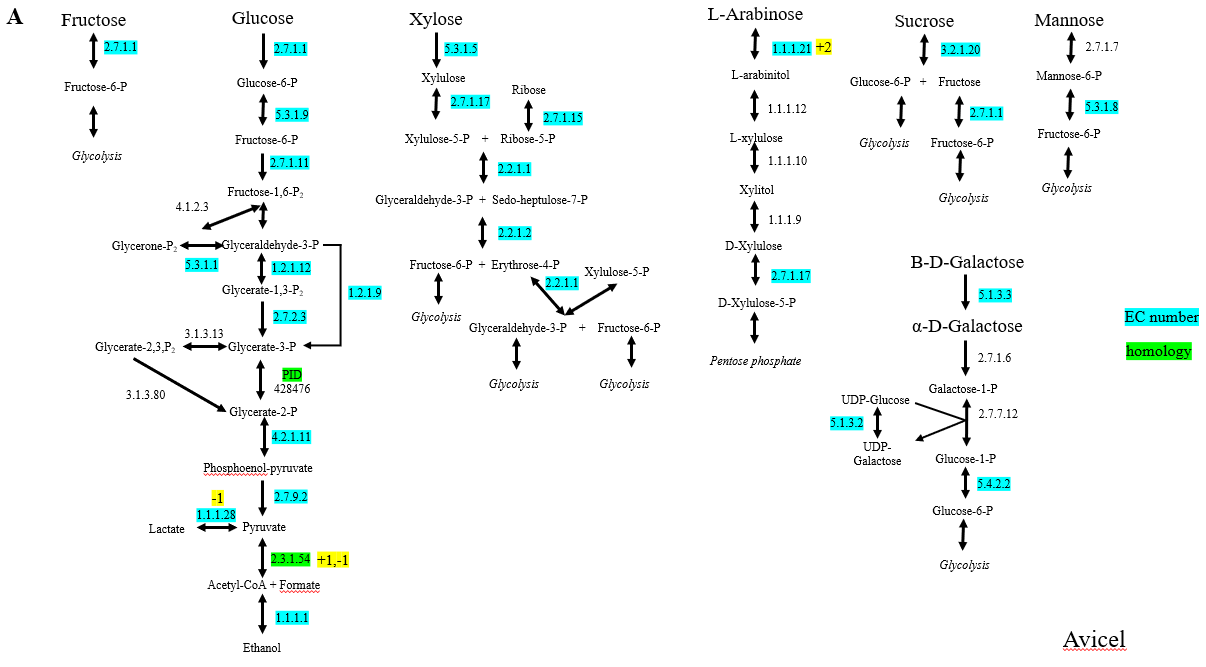


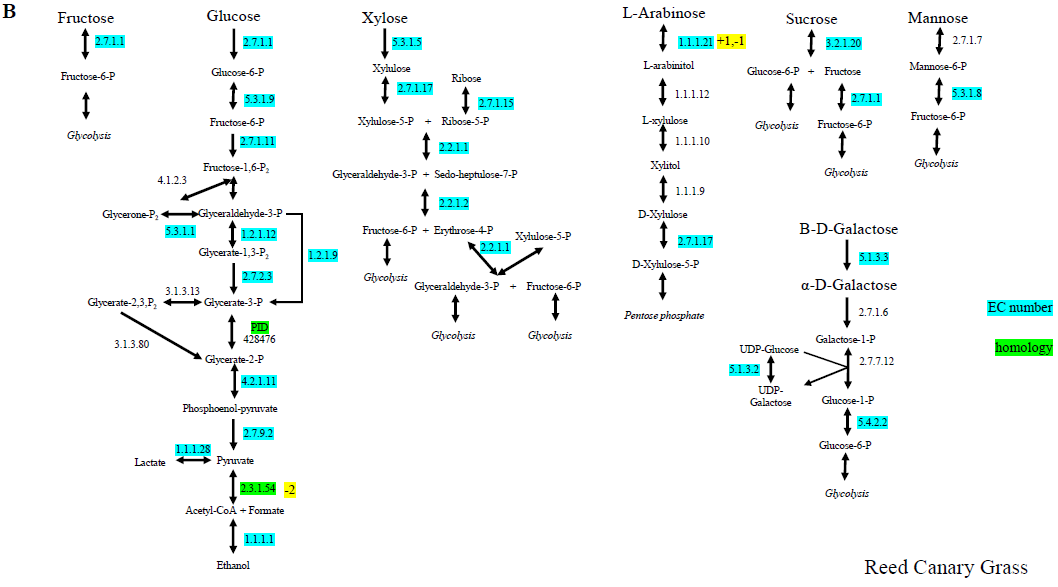


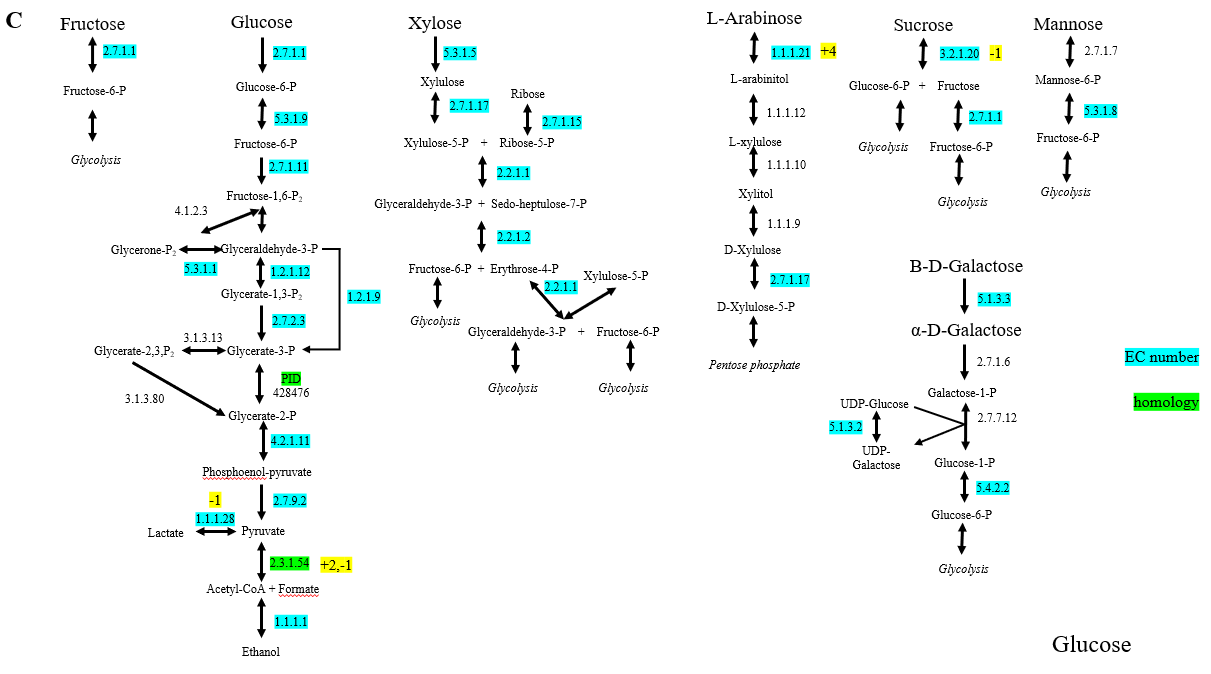


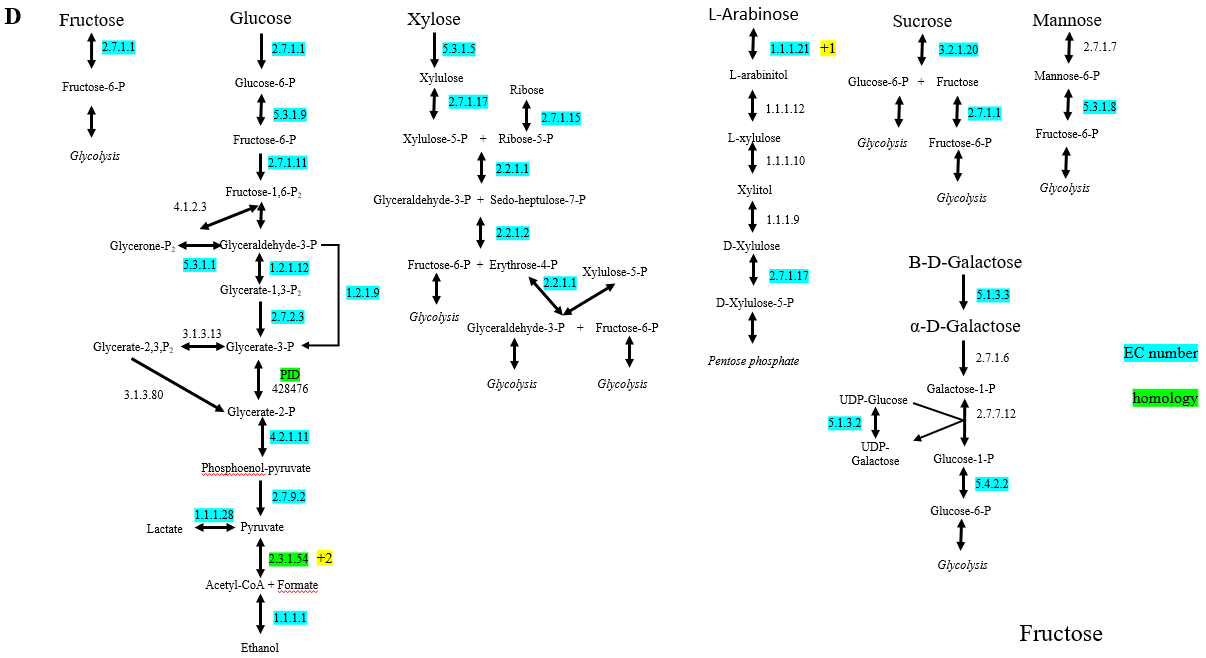


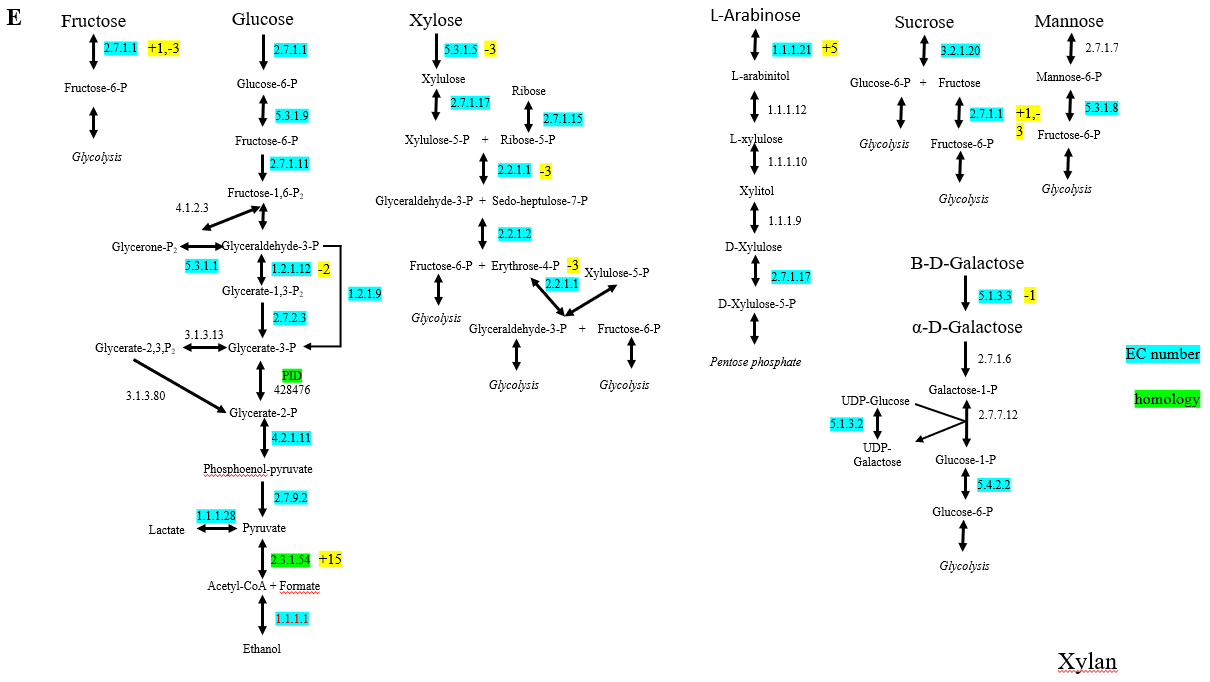


**Supplementary Figure 6. Transcriptional regulation of genes within sugar pathways for co-culture versus monoculture of *C. churrovis* and *C. churrovis* paired with *M. bryantii*.** The number of genes upregulated (+) or downregulated (-) that are annotated as each enzyme are highlighted in yellow. Enzymes were either identified by Enzyme Commission (EC) number (highlighted in blue) or homology (highlighted in green). Cultures grown on all substrates with the exception of reed canary grass had at least one gene annotated as a pyruvate formate lyase (PFL) upregulated. Results indicated that co-culture with a methanogen may enhance PFL function in cultures grown on fructose and xylan. PFLs were identified through homology to PFLs identified as hydrogenosome components in the *N. lanati* genome. Select enzymes in sugar pathways were upregulated in co-culture for some substrates, indicating enhanced production of bottleneck enzymes in sugar pathways. This figure depicts regulation in the cultures grown on Avicel^®^ (A) reed canary grass (B), glucose (C), fructose (D), and xylan (E) substrates.

|  |  |  |
| --- | --- | --- |

|  |  |
| --- | --- |
